# *Pseudomonas aeruginosa* isolates in severe chronic obstructive pulmonary disease: characterization and risk factors

**DOI:** 10.1186/1471-2466-14-103

**Published:** 2014-06-26

**Authors:** Miguel Gallego, Xavier Pomares, Mateu Espasa, Eva Castañer, Mar Solé, David Suárez, Eduard Monsó, Concepción Montón

**Affiliations:** 1 Department of Respiratory Medicine, Hospital de Sabadell, Institut Universitari Parc Taulí-UAB, Sabadell, Spain; 2 Universitat Autonoma de Barcelona, Esfera UAB, Barcelona, Spain; 3 CIBER de Enfermedades Respiratorias, CIBERES, Bunyola, Spain; 4 Department of Microbiology, SDI UDIAT, Institut Universitari Parc Taulí-UAB, Sabadell, Spain; 5 Department of Radiology, SDI UDIAT, Institut Universitari Parc Taulí-UAB; 6 Barcelona Centre for International Health Research (CRESIB), Department of Clinical Microbiology, Hospital Clínic-Fundació Clínic per la Recerca Biomèdica, University of Barcelona, Barcelona, Spain; 7 Epidemiology and Assessment Unit, Fundació Parc Taulí, Universitat Autònoma de Barcelona, Sabadell, Spain

**Keywords:** Chronic obstructive pulmonary disease, Bronchiectasis, Chronic colonization, *Pseudomonas aeruginosa*

## Abstract

**Background:**

Patients with severe chronic obstructive pulmonary disease (COPD) are at increased risk of infection by *P. aeruginosa*. The specific role of bronchiectasis in both infection and chronic colonization by this microorganism in COPD, however, remains ill defined.

To evaluate the prevalence and risk factors for *P. aeruginosa* recovery from sputum in outpatients with severe COPD, characterizing *P. aeruginosa* isolates by pulsed-field gel electrophoresis (PFGE) and focusing on the influence of bronchiectasis on chronic colonization in these patients.

**Methods:**

A case-cohort study of 118 patients with severe COPD attended at a Respiratory Day Unit for an acute infectious exacerbation and followed up over one year. High-resolution CT scans were performed during stability for bronchiectasis assessment and sputum cultures were obtained during exacerbation and stability in all patients. *P. aeruginosa* isolates were genotyped by PFGE. Determinants of the recovery of *P. aeruginosa* in sputum and chronic colonization by this microorganism were assessed by multivariate analysis.

**Results:**

*P. aeruginosa* was isolated from 41 of the 118 patients studied (34.7%). Five of these 41 patients (12.2%) with *P. aeruginosa* recovery fulfilled criteria for chronic colonization. In the multivariate analysis, the extent of bronchiectasis (OR 9.8, 95% CI: 1.7 to 54.8) and the number of antibiotic courses (OR 1.7, 95% CI: 1.1 to 2.5) were independently associated with an increased risk of *P. aeruginosa* isolation. Chronic colonization was unrelated to the presence of bronchiectasis (p=0.75). In patients with chronic colonization the isolates of *P. aeruginosa* retrieved corresponded to the same clones during the follow-up, and most of the multidrug resistant isolates (19/21) were harbored by these patients.

**Conclusions:**

The main risk factors for *P. aeruginosa* isolation in severe COPD were the extent of bronchiectasis and exposure to antibiotics. Over 10% of these patients fulfilled criteria for chronic colonization by *P. aeruginosa* and showed clonal persistence, independently of the presence of bronchiectasis.

## Background

Infectious exacerbations are the most important cause of hospital admission and mortality in severe chronic obstructive pulmonary disease (COPD) [1,2]. Potentially pathogenic microorganisms (PPMs) such as *Haemophilus influenzae, Streptococcus pneumoniae*, *Moraxella catarrhalis* and viruses are the leading causative agents of these acute episodes, but *P. aeruginosa* accounts for 5-10% of COPD exacerbations in these patients and has been associated with increased mortality [3].

It is widely accepted that the probability of bacterial infection is higher in exacerbations appearing in severe COPD patients. Two studies [4,5] have shown that individuals with a FEV1 below 50% of the reference level have a six-fold higher risk of suffering exacerbations due to *H. influenzae* and *P. aeruginosa* than patients with mild or moderate COPD. Likewise, *P. aeruginosa* infection in COPD has also been related to risk factors such as previous hospital admissions and use of oral corticosteroids or antibiotics [6-9] but the importance of these individual determinants has not been elucidated. Moreover, the role of other potential factors such as bronchiectasis, as a risk factor of *P. aeruginosa* in cursive infection-a factor associated with severe airflow obstruction in COPD [10], has been addressed only marginally [8]. In the context of COPD exacerbation, the majority of guidelines recommend specific treatment against *P. aeruginosa* in patients reporting hospital admissions and/or recurrent use of antibiotics or corticosteroids [11,12]; but the management of patients with repeated isolates of this PPM is not well established. Recent studies based on pulsed-field gel electrophoresis (PFGE) and polymerase chain reaction have shown that most clones are cleared over time and that only a minority persist [13]. In some patients a pattern of chronic colonization by *P. aeruginosa* has been reported, closely resembling those seen in cystic fibrosis [14,15]. Unfortunately, the relationship between chronic colonization by *P. aeruginosa*, the pattern of clonal carriage and bronchiectasis has not been evaluated to date.

The objectives of our study were to evaluate the prevalence and risk factors for, *P. aeruginosa* isolation in sputum samples from outpatients with severe COPD, characterizing *P. aeruginosa* isolates in these patients with pulsed-field gel electrophoresis (PFGE) and focusing on the influence of bronchiectasis and other risk factors, on bronchial colonization in these patients.

## Methods

### Study subjects

Studied patients were part of a cohort of severe COPD outpatients attending regularly Respiratory Diseases Day Care Unit of Sabadell University hospital for scheduled and exacerbation visits. This cohort included COPD patients with a postbronchodilatador FEV1 below 50% from the reference who reported three or more severe exacerbations in the previous year. Patients in the cohort received education on self-care of the disease and a personal action plan, which included unscheduled visits to the Unit when exacerbation symptoms appear.

Patients with severe COPD attending the Unit for an exacerbation of COPD (ECOPD), defined by the presence of two or three of Anthonisen’s criteria [16], were enrolled in this case-cohort study between January 2005 and March 2008, and followed from that episode onwards for a minimum period of one year.

*Exclusion criteria*: patients previously diagnosed with bronchiectasis in view of symptoms and chest X-rays, patients <40 years of age, patients diagnosed with asthma, cystic fibrosis or active neoplasia, those receiving chronic treatment with oral corticosteroids or immunosuppressive drugs for any reason and those patients under long-term systemic or inhaled antibiotic therapy.

For the purpose of the study, patients were divided into two groups according to the presence of *P. aeruginosa* in sputum samples. Patients with one or more isolates of *P. aeruginosa* from sputum at baseline or during the follow-up, or with *P. aeruginosa* recoveries from sputum samples in the previous year were assigned to the *P. aeruginosa* group (PA group), and patients with no previous recovery of *P. aeruginosa* and who did not present this microorganism from their sputum samples during the follow-up were assigned to the non-*P. aeruginosa* group (non-PA group). Written informed consent was obtained from each subject and ethical permission for the study was obtained from the Sabadell University Hospital Ethics Committee.

### COPD clinical evaluation

COPD was defined as a postbronchodilator ratio of FEV_1_ to FVC of less than 0.7 according to the Global Initiative for Chronic Obstructive Lung Disease (GOLD) criteria in a patient with a smoking habit of more than 10 pack-years [12]. At baseline, smoking history, severity of the disease, BODE index [17], comorbidities (Charlson index) [18], use of long-term home oxygen therapy, short-term treatments with antibiotics and corticosteroids and hospitalizations due to respiratory causes in the previous year were recorded.

All patients underwent the same scheduled clinical controls at the Respiratory Day Care Unit [19] by the same team of pulmonologists and received regular treatment with long-term beta-agonists (LABA), anticholinergics and inhaled corticosteroids (ICS). After the enrolment visit, scheduled visits were recorded together with unscheduled acute ECOPD visits, hospitalizations and mortality due to respiratory causes. When acute symptoms appeared, chest radiography was performed to rule out acute pneumonia and decisions regarding antibiotic treatment and admission were left to the discretion of attending physicians in accordance with international guidelines [11,12], adjusting the antibiotic prescriptions to the antibiogram. In case of *Pseudomonas aeruginosa* isolation, oral quinolone (ciprofloxacin or levofloxacin) or anti-pseudomonal intravenous therapy was initiated according to the severity of the episode.

Isolation of *P. aeruginosa* in three or more consecutive sputum cultures, taken at least one month apart during a 6-month period, was considered as chronic colonization by this PPM, independently of their recovery from exacerbation or stability periods and assuming the persistence of the microorganism in the periods of stability between exacerbations, [20]. In these patients, chronic treatment with inhaled colistin plus long-term azithromycin was indicated and prescribed from the diagnosis.

### Bronchiectasis assessment

CT scans were performed in stability to evaluate bronchiectasis using a Multidetector CT scanner (Sensation 16 Siemens; Erlangen, Germany). High resolution CT (HRCT) images were obtained, during fully suspended inspiration in the supine position from the lung apex to the diaphragm, using a thin-section technique (1-mm collimation at 10-mm intervals) with the following parameters: 120 kVp tube voltage, 250 mA, and 1-second scanning time. The images were obtained without injection of contrast material and viewed in a lung window (window width, 1500 HU; window level, âˆ’600 HU), using a high spatial frequency algorithm for image reconstruction.

The CT scans were interpreted for the presence of bronchiectasis by two radiologists experienced (EC and XG) in the interpretation of HRCT and blinded to the patient grouping. Any differences in the reading were resolved by consensus. Presence of bronchiectasis was based on the following criteria: non-tapering bronchus with internal diameter 110% or greater than the adjacent pulmonary artery, or visible bronchi within 1 cm of the costal or mediastinal pleural surface [21]. Bronchiectasis extent was scored for each pulmonary lobe, grading the lingula as a separate lobe. Small bronchiectasis only visible in a single pulmonary segment were not considered, as they may appear in a significant percentage of healthy population [22]. The grading system proposed by Smith and coworkers [23] was used in the study, scoring the absence of bronchiectasis as 0, bronchiectasis in fewer than 25% of bronchi as 1, in 25-49% of bronchi as 2, in 50-74% as 3, and in 75% or more as 4. Total score ranged from a minimum value of 0 points corresponding to absence of bronchiectasis, to a maximum value of 24 points indicating involvement of all pulmonary lobes. Patients with a score ≤ 1 were considered as normal.

### Sputum bacteriology

Spontaneous sputum samples were collected in each ECOPD before antibiotic administration. Stability samples were collected in scheduled visits at least one month after the clinical resolution of any previous acute episode in patients producing spontaneous sputum. Recovered sputum was processed within 60 minutes of collection in all cases, and Murray-Washington criteria were used for the identification and selection of samples representative of bronchial secretions [24]. Sputum samples graded Murray IV-V were cultured for PPMs in selective media according to standard methods [25], and cultures were considered positive when PPMs were recovered.

Briefly, samples were vortexed for 30–60 seconds after homogenization with sputolysin and 10 uL were cultured using a calibrated loop in blood agar, blood agar with nalidixic acid, chocolate agar and McConkey agar plates. All plates were incubated in a 5% carbon dioxide incubator at 35°C and read at 18 and 48 hours. Gram-negative and Gram-positive bacteria recognized as agents causing respiratory infections, such as *H. influenzae*, *Haemophilus parainfluenzae*, *S.pneumoniae*, *M. catarrhalis*, *P. aeruginosa*, *Staphylococcus aureus*, and Enterobacteriaceae were considered as PPMs [26]. The presence or absence of a mucoid phenotype was recorded for each isolate. Antimicrobial susceptibility testing of *P. aeruginosa* isolates were performed using the microdilution assay to determine the minimum inhibitory concentration according to the procedures of the Clinical and Laboratory Standards Institute [27]. Multidrug resistance (MDR) in *P. aeruginosa* was defined as resistance to three or more antibiotic groups usually active against the PPM, including beta-lactams (penicillins and cephalosporins), carbapenems, quinolones or aminoglycosides, monobactam and polymyxins [28].

### *P. aeruginosa* genotyping

*P. aeruginosa* isolates were frozen at -80°C and subsequently genotyped. The genetic relatedness of the *P. aeruginosa* isolates in the present study was assessed by pulsed-field electrophoresis (PFGE), essentially following a modification of the method of Durmaz et al. [29]. Genomic DNA was prepared after centrifugation of 1 ml from a 1.5 McFarland solution obtained from fresh cultures grown on blood agar plates. Pellets were resuspended in 120 μL of suspension buffer (CSB; 100 mM Tris–HCl [pH 8.0], 10 mM EDTA) and mixed with an equal volume of molten (55°C) 2% InCert^®^ agarose (Lonza) prepared in CSB with 1% sodium dodecyl sulphate (SDS). The DNA was digested with30 U of SpeI restriction enzyme (New England Biolabs) and incubated at 37°C for 17 hours. The digested DNA was electrophoresed in a CHEF-DR III system (Bio-Rad) with the following running parameters: 6 V/cm at 14°C with pulse times ranging from 5 to 25 seconds for 20 hours. Gels were stained with SYBR^®^ Safe (Invitrogen) and documented in a ImageQuant™ LAS 4000 (GE Healthcare). Gel images were analyzed using InfoQuest™ FP software version 4.5 (Bio-Rad, Laboratories). Cluster analysis was generated using the Dice coefficient, with an unweighted pair-group method that uses arithmetic average (UPGMA) clustering, with a tolerance setting of 1% and optimization of 0.5%. Isolates were defined as the same PFGE clonal type if the Dice coefficient was ≥90%.

### Statistical analysis

The SPSS statistical package version 17.0 (SPSS; Chicago, Illinois) was used for the statistical analysis. Results for categorical variables were expressed as absolute and relative frequencies, and continuous variables were expressed as means and standard deviations (SD). PA and non-PA groups were compared for the assessment of factors associated with *P. aeruginosa* isolation, using the chi-square test with continuity correction for categorical variables. Quantitative variables were analyzed using Student’s t-test or the corresponding non-parametrical tests when required.

Multivariate analysis using stepwise logistic regression was performed with PA isolation as dependent variable. Variables showing a univariate association (p<0.10) were included in the model as covariates. Multicollinearity was evaluated by means of Variance Inflation Factor (VIF); considering that values below 2.5 in each covariate ruled out collineality in the model. Results were expressed as crude and adjusted odds ratios (OR) with 95% confidence intervals (95% CI). All statistical tests were two-sided, with a p value of 0.05 or less reported as statistically significant.

## Results

### Population sample

One hundred eighteen severe COPD patients with a mean (SD) follow-up of 1003 (306) days were enrolled over the study period. The study population was predominantly male, with a mean (SD) age of 69 (8) years, and classified as GOLD IV in over half of the cases. Clinical characteristics of participating patients at baseline are shown in Table 1.

**Table 1:** **Baseline and clinical characteristics of 118 severe COPD patients attended for COPD exacerbation (ECOPD), with and without *P. aeruginosa *** **isolation**

	**Whole group**	**PA-group**	**Non PA-group**	**P value**
**Subjects, (%)**	118	41 (34.7)	77 (65,3)	â€¦
**Follow-up (days), m (SD)**	1003±306	1036.9±347.5	984.9±282.3	0.41
**Age (years), m (SD)**	69.5±8.2	70.3±7.7	69±8.5	0.42
**Current smoking, (%)**	13 (11)	2 (4.9)	11 (14.3)	0.21
**Pack-year, m (SD)**	63.3±31,9	69.4±36.3	60±28.9	0.15
**Alcohol abuse >80 g/l, n (%)**	3 (2.5)	1 (2.4)	2 (2.6)	1
**Influenza vaccination, n (%)**	105 (89)	38 (92.7)	67 (87)	0.53
**Pneumococcus vaccination n (%)**	57 (48.3)	23 (56.1)	34 (44.2)	0.21
**Body mass index (Kgr/m**^**2**^**)**	27.5±4.6	26.5±4	28.1±4.9	0.17
**FEV1 post-BD, (L), m (SD)**	0.97±0.3	0.91±0.28	1.0±0.31	0.13
**FEV1 post-BD, (% predicted), m (SD)**	34±11	34±13	34.3±10	0.82
**GOLD stage IV, n (%)**	71 (60)	27 (66)	44 (57)	0.35
**BODE score, m (SD)**	5.1±1,6	5.3±1,5	5±1.6	0.38
**Home oxygen therapy, n (%)**	50 (42.4)	21 (51.2)	29 (37.7)	0.15
**Charlson, m (SD)**	4.13±1.5	4.29±1.5	4.04±1.5	0.39
**Bronchiectasis score, m (SD)**	2.1±2,6	3±3.5	1.6±1.8	**0.02**
**ECOPD during follow-up, m (SD)**	3.19±2.9	4.4±4	2.5±2	**0.008**
**ECOPD/year, m (SD)**	2.19±2	3±2.7	1.7±1.4	**0.006**
Antibiotic prescriptions*	8.9±7.5	12.5±9.2	7.01±5.7	**<0.001**
**Antibiotic prescriptions/year**	3.3±2.5	4.7±2.9	2.6±1.8	**<0.001**
Corticosteroid courses*	6.3±5.8	8±6.3	5.5±5.4	**0.02**
**Corticosteroid courses/year**	2.30±1.8	2.9±2	1.9±1.6	**0.004**
Days of hospital stay*	29.4±37.9	43.8±43.9	21.3±31.9	**0.005**
**Mortality, n (%)**	17 (14.4)	11 (27)	6 (8)	**0.005**

Data are presented as means±SD or n (%), unless otherwise stated. PA group=*P. aeruginosa group;* Non PA group=Non *P. aeruginosa* group; ns=not significant; post-BD=post bronchodilator. *prior year included.

A total of 466 sputum samples were obtained at baseline and during the follow-up, both during exacerbations (n=386) and in stability (n=80). Three hundred and twenty PPMs were isolated from 263 sputum samples and in 92 sputum samples no PPM was recovered. The most frequently isolated bacteria were *H. influenzae* (94 recoveries, 29.3%) *P. aeruginosa *(92, 28.7%) *S. pneumoniae* (51, 15.9%) and *M. catarrhalis* (46, 14.3%).

### Prevalence and risk factors for *P. aeruginosa* isolation

*P. aeruginosa* was recovered once or more from sputum samples in 41 of 118 patients (34.7%) who were included in the PA group. In five of them, isolates were reported during the year before inclusion with posterior negative samples for this PPM during the follow-up period; twenty-two had isolates growing *P. aeruginosa* before and during study period, and in the remaining 14 patients, this PPM was recovered only during follow-up.

Group comparison showed no significant differences in age, smoking history, FEV1, body mass index, BODE score, comorbidities, influenza and pneumococcus vaccination and long-term oxygen therapy use. Patients in the PA group had significantly more COPD exacerbations, higher antibiotic and corticosteroid prescription rates, and longer hospital stays than the non-PA group (Table 1).

Bronchiectasis were found in 56 patients (47%), with a mean score of 4.2 (2.5) when present. In 29 patients (51.8%) bronchiectasis were localized only in lower lobes, and in 12 participants (25.4%) were identified in more than four lobes. Bronchiectasis scores were significantly higher in the PA group than in the non-PA group (3 vs 1.6, p=0.02). Mortality was also significantly higher in the PA group than in the non-PA group (27% vs 8%, p=0.005) (Table 1).

For the identification of risk factors for *P.aeruginosa* isolation in severe COPD, bronchiectasis were categorized in three groups according to their extent: 1) No bronchiectasis; 2) bronchiectasis scored 2–5; and 3) bronchiectasis scored over 5, using the first group as reference category. Forty-five patients (38%) had scores between 2 and 5, and 11 patients (9%) had more extensive bronchiectasis with scores higher than 5. Multivariate analysis showed that the extent of bronchiectasis (OR 9.8, 95% CI: 1.7 to 54.8) and the number of courses of antibiotic therapy (OR 1.7, 95% CI: 1.1 to 2.5) were independently associated with an increased risk of *P. aeruginosa* isolation in bronchial secretions (Table 2).

**Table 2:** **Results of multivariate analysis of factors associated with *P. aeruginosa *** **isolation**

**Factor**	**OR**	**95% CI**	**P value**
Bronchiectasis score (>5)	**9.8**	**1.7 - 54.8**	**0.009**
Antibiotic prescriptions/year	**1.7**	**1.1 – 2.5**	**0.008**
Days of hospital stay	1	0.9 - 1	0.3
Corticosteroid courses/year	0.7	0.5 – 1.2	0.2

### *P. aeruginosa* colonization and bronchiectasis

Five of 41 patients (12.2%) fulfilled criteria of chronic colonization by P. aeruginosa without a relationship between this clinical situation and the extent of bronchiectasis. Three out of five chronically colonized patients (60%) did not show bronchiectasis on the CT scan; nor did 15 out of 23 patients who had single isolates of *P. aeruginosa* (65%) (p=0.75). The observation of a mucoid morphotype, however, was significantly associated with a bronchiectasis score over 5 (p=0.004), and found in four out of five patients with this morphotype.

### Characterization of *P. aeruginosa* isolates

**Figure 1:**
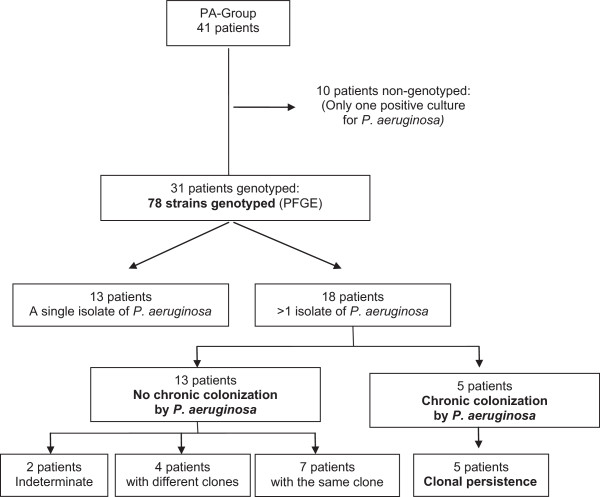
**Flow chart illustrating the distribution of PA-group patients with *P. aeruginosa *isolates genotyped.** The indeterminate group corresponds to patients with three isolates, but only one isolate genotyped.

**Figure 2:**
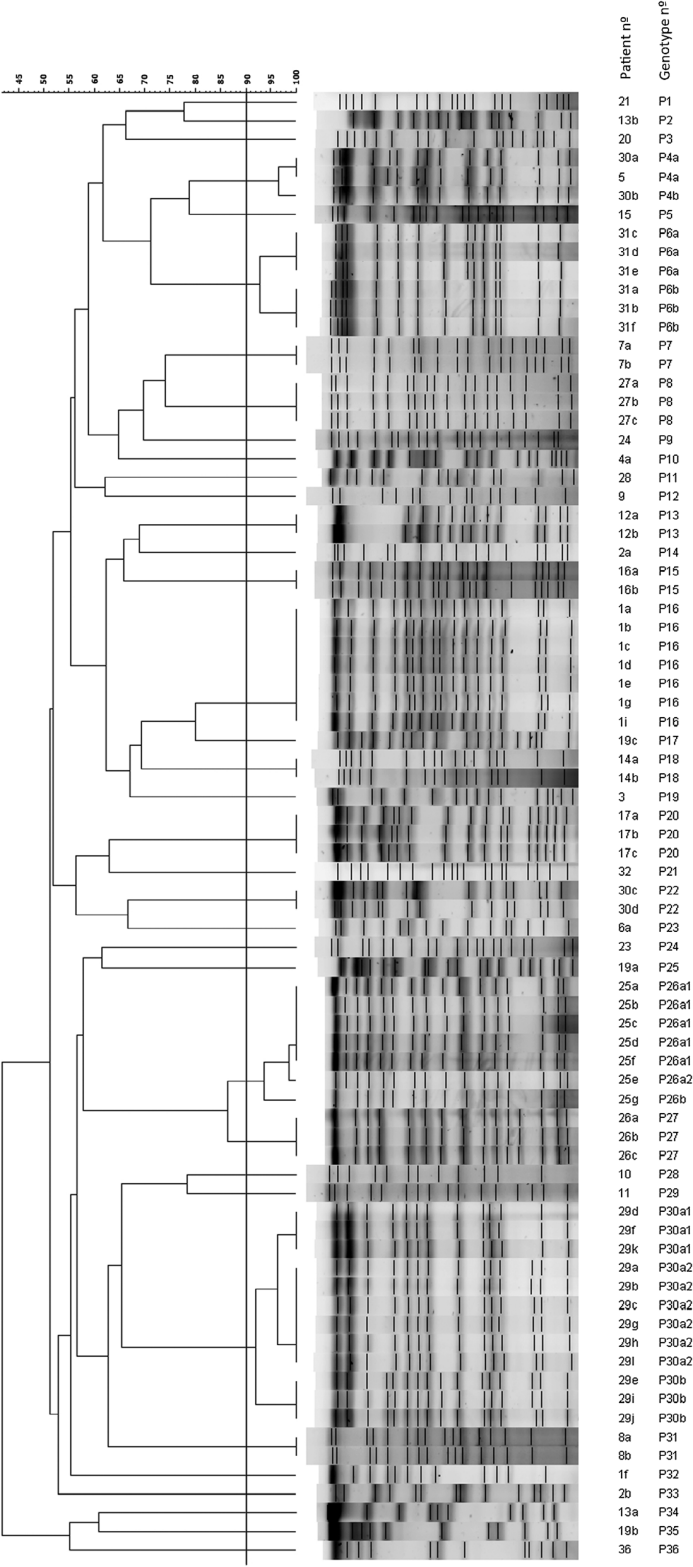
**Dendogram of PFGE patterns for 78 *P. aeruginosa *isolates.** A genetic similarity index scale is shown above the dendrogram. The line in the dendrogram denotes the homology threshold of 90% used for defining groups of genetic similarity.

Ninety-two positive cultures of *P. aeruginosa* were recovered from 41 patients, with 78 isolates valid for genotyping, corresponding to 31 patients. Thirty-six clones were identified from these 78 isolates, specific for each patient, except in the case of one clone shared by two patients. Figure 1 shows the overall scheme of the PA group with *P. aeruginosa* genotyped, and Figure 2 shows the dendogram of PFGE patterns for the 78 genotyped *P. aeruginosa* isolates.

Thirteen of 31 patients (41.9%) showed a single *P. aeruginosa* isolate with a single clone. Among the remaining 18 patients with repeated isolates of *P. aeruginosa*, 12 patients showed the same clone during follow-up, five of them fulfilling the criteria for chronic colonization. The assessment of mucoid morphotype in the genotyped samples demonstrated this morphotype in 11 isolates from five different patients (numbers 1, 25, 28, 30, and 36; in Tables 3 and 4) with three clones (P16, P26 and P4) adopting different morphotypes over time (Tables 3 and 4).

**Table 3:** **Characteristics of *P. aeruginosa *** **isolates genotyped in patients who met criteria for chronic bronchial colonization by *P. aeruginosa***

**Patient isolates**	**PFGE Genotype**	**ECOPD**	**Isolation date**	**Morphotype**
1e	P16	No	24/Mar/06	*Pseudomonas aeruginosa*
1f	P32	No	24/Mar/06	*Pseudomonas aeruginosa*
1a	P16	Yes	10/Jan/07	*Pseudomonas aeruginosa*
1b	P16	No	16/Feb/07	*Pseudomonas aeruginosa*
1g	P16	Yes	21/Mar/07	*Pseudomonas aeruginosa*
1i	P16	Yes	21/Mar/07	*Pseudomonas aeruginosa* mucoid
1c	P16	Yes	23/Apr/07	*Pseudomonas aeruginosa* mucoid
1d	P16	Yes	31/May/07	*Pseudomonas aeruginosa*
25a	P26^a1^	Yes	18/Aug/05	*Pseudomonas aeruginosa* mucoid
25b	P26^a1^	No	3/Nov/05	*Pseudomonas aeruginosa* mucoid
25c	P26^a1^	Yes	5/Dec/05	*Pseudomonas aeruginosa*
25d	P26^a1^	No	9/Jan/06	*Pseudomonas aeruginosa* mucoid
25e	P26^a2^	No	17/Mar/06	*Pseudomonas aeruginosa* mucoid
25f	P26^a1^	Yes	11/Jan/07	*Pseudomonas aeruginosa* mucoid
25g	P26^b^	Yes	23/Apr/07	*Pseudomonas aeruginosa* mucoid
26a	P27	Yes	10/Oct/07	*Pseudomonas aeruginosa*
26b	P27	Yes	12/Nov/07	*Pseudomonas aeruginosa*
26c	P27	No	14/Dec/07	*Pseudomonas aeruginosa*
29a	P30^a2^	Yes	2/Mar/05	*Pseudomonas aeruginosa*
29b	P30^a2^	No	17/Jun/05	*Pseudomonas aeruginosa*
29c	P30^a2^	Yes	9/Nov/05	*Pseudomonas aeruginosa*
29k	P30^a1^	Yes	12/Dec/05	*Pseudomonas aeruginosa*
29l	P30^a2^	Yes	12/Dec/05	*Pseudomonas aeruginosa*
29d	P30^a1^	No	23/Jan/06	*Pseudomonas aeruginosa*
29e	P30b	No	3/Jul/06	*Pseudomonas aeruginosa*
29f	P30^a1^	No	18/Sep/06	*Pseudomonas aeruginosa*
29g	P30^a2^	No	7/Nov/06	*Pseudomonas aeruginosa*
29h	P30^a2^	No	27/Dec/06	*Pseudomonas aeruginosa*
29i	P30^b^	No	12/Sep/07	*Pseudomonas aeruginosa*
29j	P30^b^	No	17/Dec/07	*Pseudomonas aeruginosa*
31a	P06^b^	Yes	9/Oct/06	*Pseudomonas aeruginosa*
31b	P06^b^	No	21/Nov/06	*Pseudomonas aeruginosa*
31c	P06^a^	Yes	22/Mar/07	*Pseudomonas aeruginosa*
31d	P06^a^	No	27/Apr/07	*Pseudomonas aeruginosa*
31e	P06^a^	Yes	4/Sep/07	*Pseudomonas aeruginosa*
31f	P06^b^	No	17/Oct/07	*Pseudomonas aeruginosa*

Patients are identified by numbers and different isolates by letters. The PFGE clones are identified by numbers and the superscript indicates same clones with a Dice coefficient between 90-99%.

**Table 4:** **Characteristics of *P. aeruginosa *** **isolates genotyped in patients without criteria for chronic bronchial colonization by *P. aeruginosa***

**Patient isolates**	**PFGE Genotype**	**ECOPD**	**Isolation date**	**Morphotype**
2a	P14	Yes	17/Oct/06	*Pseudomonas aeruginosa*
2b	P33	Yes	15/Feb/08	*Pseudomonas aeruginosa*
3	P19	Yes	17/Oct/05	*Pseudomonas aeruginosa*
4	P10	Yes	2/Jun/05	*Pseudomonas aeruginosa*
5	P04^a^	Yes	22/Mar/06	*Pseudomonas aeruginosa*
6	P23	Yes	4/Jul/05	*Pseudomonas aeruginosa*
7a	P07	Yes	1/Dec/06	*Pseudomonas aeruginosa*
7b	P07	No	12/Nov/07	*Pseudomonas aeruginosa*
8a	P31	No	8/Sep/05	*Pseudomonas aeruginosa*
8b	P31	No	12/Dec/05	*Pseudomonas aeruginosa*
9	P12	Yes	14/Dec/07	*Pseudomonas aeruginosa*
10	P28	Yes	10/Aug/06	*Pseudomonas aeruginosa*
11	P29	Yes	5/Oct/07	*Pseudomonas aeruginosa*
12a	P13	Yes	7/Apr/05	*Pseudomonas aeruginosa*
12b	P13	No	18/Oct/05	*Pseudomonas aeruginosa*
13a	P34	Yes	30/May/06	*Pseudomonas aeruginosa*
13b	P02	Yes	11/Oct/06	*Pseudomonas aeruginosa*
14a	P18	Yes	28/Jan/06	*Pseudomonas aeruginosa*
14b	P18	Yes	28/Jul/06	*Pseudomonas aeruginosa*
15	P05	Yes	2/Feb/06	*Pseudomonas aeruginosa*
16a	P15	Yes	23/Nov/05	*Pseudomonas aeruginosa*
16b	P15	Yes	3/Jan/06	*Pseudomonas aeruginosa*
17a	P20	Yes	3/Nov/05	*Pseudomonas aeruginosa*
17b	P20	Yes	22/Dec/05	*Pseudomonas aeruginosa*
17c	P20	Yes	8/Jun/06	*Pseudomonas aeruginosa*
19a	P25	Yes	19/Dec/05	*Pseudomonas aeruginosa*
19b	P35	No	12/Jun/06	*Pseudomonas aeruginosa*
19c	P17	Yes	3/Mar/08	*Pseudomonas aeruginosa*
20	P03	Yes	28/Nov/06	*Pseudomonas aeruginosa*
21	P01	Yes	4/Feb/08	*Pseudomonas aeruginosa*
23	P24	Yes	6/Mar/06	*Pseudomonas aeruginosa*
24	P09	Yes	30/Jun/06	*Pseudomonas aeruginosa*
27a	P08	Yes	20/Mar/06	*Pseudomonas aeruginosa*
27b	P08	No	12/Apr/06	*Pseudomonas aeruginosa*
27c	P08	No	25/Jul/07	*Pseudomonas aeruginosa*
28	P11	Yes	11/Oct/07	*Pseudomonas aeruginosa* mucoid
30a	P04^a^	Yes	10/Jan/07	*Pseudomonas aeruginosa*
30b	P04^b^	No	21/Feb/07	*Pseudomonas aeruginosa* mucoid
30c	P22	No	19/Dec/07	*Pseudomonas aeruginosa*
30d	P22	Yes	8/Feb/08	*Pseudomonas aeruginosa*
36	P36	Yes	23/May/05	*Pseudomonas aeruginosa* mucoid
32	P21	Yes	10/Nov/06	*Pseudomonas aeruginosa*

Patients are identified by numbers and different isolates by letters. The PFGE clones are identified by numbers and the superscript indicates same clones with a Dice coefficient between 90-99%.

Thirty-seven isolates of *P. aeruginosa* analyzed were susceptible to all tested antibiotics (47.4%), 20 were resistant to one or two antibiotic groups (25.6%), while the remaining 21 were multidrug resistant (26.9%). The antibiotic resistance profile of the 78 genotyped *P. aeruginosa* isolates is shown in Table 5. Multidrug resistant isolates were retrieved in five patients (numbers 1, 13, 19, 25 and 29; in Tables 3 and 4), three of them chronically colonized. These three patients harbored 19 of the 21 multidrug resistant isolates observed (90%). The clones of all these patients showed fluctuations in antimicrobial susceptibility over time.

**Table 5:** **Antibiotic resistance profile of 78 *P. aeruginosa *** **isolates**

**Antibiotic group**	**Antibiotic**	**Number of susceptible strains (%)**
**Quinolones**		39 (50.0)
	Ciprofloxacin	37 (47.4)
	Levofloxacin	39 (50.0)
**Aminoglycosides**		63 (80.8)
	Tobramycin	54 (69.2)
	Amikacin	61 (78.2)
	Gentamicin	37 (47.4)
**Penicillins**		63 (80.8)
	Ticarcillin	58 (74.4)
	Piperacillin tazobactam	63 (80.8)
**Cefalosphorins**		58 (74.4)
	Ceftazidime	58 (74.4)
	Cefepime	51 (65.4)
**Carbapenems**		62 (79.5)
	Imipenem	50 (64.1)
	Meropenem	62 (79.5)
**Polymyxins**	Colistin	76 (97.4)
**Monobactams**	Aztreonam	59 (75.6)

## Discussion

Our study showed a high prevalence of P*. aeruginosa* isolation in a cohort of severe COPD patients. The extent of bronchiectasis and recurrent use of antibiotic therapy were the main risk factors for the recovery of this PPM from bronchial secretions. Most patients in the PA group had single isolates of *P. aeruginosa,* but up to 12% of patients met criteria for chronic colonization by this PPM, though this clinical situation was not related to the presence of bronchiectasis. Furthermore, clonal persistence was observed in this subset of patients, as previously reported in patients with cystic fibrosis.

### Prevalence and risk factors for *P. aeruginosa* isolation

We recovered *P. aeruginosa* from sputum in nearly 40% of the severe COPD patients in our series, half of whom presented bronchiectasis. These figures were similar to those reported in previous studies in patients with advanced disease, as shown by Murphy et al. [30] and Renom et al. [9], who reported *P. aeruginosa* isolation in over one third of the sputum samples obtained from COPD patients.

A strong relationship between the extent of bronchiectasis and *P. aeruginosa* recovery was observed in our study. Bronchiectasis have been often reported in severe COPD [10,21,31] but the association between bronchiectasis and *P. aeruginosa* isolation have been only marginally addressed in previous studies. García-Vidal et al. [8] analyzed 188 patients with moderate to severe COPD admitted to hospital for a COPD exacerbation and studied by HRCT identifying bronchiectasis in 52% of them, without finding significant relationships between bronchiectasis and *P. aeruginosa* isolation. In a similar population of 201 COPD patients Martínez-García et al. [22] observed that the isolation of *P. aeruginosa* was significantly more frequent in patients with bronchiectasis (13% versus 4.7%). Our study demonstrated that in COPD patients with advanced disease the relationship between bronchiectasis and the recovery of *P. aeruginosa* is clearly defined and associated with the extent of the bronchial abnormality. Additionally, among the other risk factors evaluated for *P. aeruginosa* isolation in our study, only the number of previous antibiotic treatments was independently associated with the recovery of this PPM in our study, after accounting for covariates in the multivariate analysis. This finding is in agreement with previous studies that have found a relationship between antibiotic treatments and the subsequent isolation of *P. aeruginosa* in sputum samples [7,8]. The lack of a relationship in the multivariate analysis between previous hospital admissions and *P. aeruginosa* recovery in our study may be attributed to the differences in study design, because the present study focus on patients regularly attending an outpatient day care facility, or to the higher weight of recurrent antibiotic treatments as a predictor. Although the present study was not designed to evaluate mortality, we observed a higher death rate during the follow-up in the PA group, in agreement with other authors [9,32].

### Characterization of *P.aeruginosa* isolates in chronic colonization

Near 50% of COPD patients (PA-group) had repeated isolates of *P. aeruginosa*, accomplishing criteria of chronic colonization by this PPM in over 10% of cases. Similarly, Martinez- García et al. [31] found chronic colonization by *P. aeruginosa* in 11/201 moderate-to-severe COPD patients. The molecular characterization of *P. aeruginosa* isolates in our COPD cohort demonstrated the persistence of the same clone in two thirds of them and in all chronically colonized patients.

Few studies to date have evaluated chronic colonization by *P. aeruginosa* in COPD patients and those that have been performed have showed conflicting results. Studies assessing *P. aeruginosa* isolates by PFGE from COPD respiratory samples showed a chronic infection pattern similar to cystic fibrosis [14,15] in a limited number of COPD patients. Conversely, in a 10-year follow-up study which included 126 COPD patients, Murphy et al. [30] observed two distinct patterns of carriage of *P. aeruginosa*: a more frequent pattern of short-term carriage followed by clearance of the strain in up to half of episodes, and a persistence pattern of the same strain in a quarter of the episodes. Rakhimova et al. [13] genotyped *P. aeruginosa* isolates from this cohort and compared them with strains recovered from patients with cystic fibrosis showing chronic *P. aeruginosa* infection (n=128), concluding that sporadic or intermittent infection with *P. aeruginosa* was the most common finding in bronchial secretions from COPD patients, who presented a frequent clone turnover, a pattern that differs from the chronic carriage of *P. aeruginosa* clones found in cystic fibrosis. Our results confirm that although the most frequent pattern in severe COPD is a self-limited isolation of *P. aeruginosa*, over ten per cent of severe patients show chronic colonization by *P. aeruginosa*, with clonal persistence pattern in close similarity with cystic fibrosis. A relationship with the extent of bronchiectasis was not observed in our study in this subset of patients, however. Determinants other than bronchiectasis that were not evaluated in our study, such as virulence factors or biofilm production, could explain the ability of *P. aeruginosa* to colonize these patients [14]. More than half of the isolates recovered from chronically colonized patients who harbor the same clone showed multidrug resistance and presented fluctuations in their antimicrobial susceptibility over the time. This variability in the antibiogram of *P. aeruginosa* has been reported previously [33] in cystic fibrosis, suggesting a polyclonal population that may not be well represented in sputum cultures. Similarly, some *P. aeruginosa* clones in our study adopted both mucoid and non-mucoid phenotypes during the follow-up, a finding that has also been reported in cystic fibrosis [34]. Finally, although the mucoid morphotype is considered a marker of colonization [35], in our study it was mainly related to high score of bronchiectasis.

### Limitations

Patients from a selected COPD population participated in this study and our results can not be extrapolated to the COPD population as a whole. Only patients with severe COPD and frequent exacerbations who were managed on an outpatient basis at a day care facility were enrolled: results in these patients may not be applicable to patients who require recurrent admissions when exacerbation symptoms appear or has less severe disease. Additionally, microbiological analyses were performed only in spontaneous sputum samples, and no information on bronchial flora was obtained from patients or from episodes without sputum production. These limitations, however, do not reduce the significance of the results obtained, considering that spontaneous sputum production is the most common situation for these patients during exacerbations.

## Conclusions

In summary, our study suggests a high prevalence of *P. aeruginosa* isolations in severe COPD patients. The extent of bronchiectasis and the number of antibiotic treatment regimens received were the main independent risk factors for the recovery of this PPM. Moreover, over one tenth of COPD patients met criteria for chronic colonization by *P. aeruginosa* and showed clonal persistence of this PPM regardless of the extent of bronchiectasis.

These findings have clinical implications for the management of severe COPD patients with frequent exacerbations, who may need the performance of CT scans and repeated sputum cultures for the identification of bronchiectasis and bronchial colonization by this PPM. *P. aeruginosa* must be considered a common causative agent in these patients, especially in those with a high extent in their bronchiectasis and receiving frequent antibiotic treatment. According to our data, a close follow-up may be recommended when *P. aeruginosa* is isolated in sputum cultures, for an early identification of chronic colonization, a situation which will require an approach similar to that applied in cystic fibrosis.

## Competing interest

All authors declare not having competing interests that might have influenced the performance or presentation of our work in this manuscript.

## Authors’ contributions

This work is original and all authors meet the criteria for authorship, including acceptance of responsibility for the scientific content of the manuscript. Conception and design of the study: MG, CM, XP, EC Collection of samples: XP, CM, ME, MS Acquisition and interpretation of data: MG, EM, DS, EC, ME Writing the article: MG, XP, CM, EM. All authors read and approved the final manuscript.

## Pre-publication history

The pre-publication history for this paper can be accessed here:

http://www.biomedcentral.com/1471-2466/14/103/prepub
